# In Vitro Collection for the Safe Storage of Grapevine Hybrids and Identification of the Presence of *Plasmopara viticola* Resistance Genes

**DOI:** 10.3390/plants13081089

**Published:** 2024-04-13

**Authors:** Natalya V. Romadanova, Moldir M. Aralbayeva, Alina S. Zemtsova, Alyona M. Alexandrova, Saule Zh. Kazybayeva, Natalya V. Mikhailenko, Svetlana V. Kushnarenko, Jean Carlos Bettoni

**Affiliations:** 1 Institute of Plant Biology and Biotechnology, 45 Timiryazev St., Almaty 050040, Kazakhstan; berim.moldir@mail.ru (M.M.A.); georgi-nata@mail.ru (N.V.M.); sv.kushnarenko.bio@gmail.com (S.V.K.); 2 M.A. Aitkhozhin Institute of Molecular Biology and Biochemistry, Almaty 050012, Kazakhstan; a.alexandrova@imbb.org.kz; 3 Kazakh Scientific Research Institute of Fruit Growing and Viticulture, Almaty 050060, Kazakhstan; saule_5_67@mail.ru; 4 Independent Researcher, 35 Brasil Correia Street, Videira 89560510, Brazil

**Keywords:** disease management, downy mildew, ex situ conservation, micropropagation, *Vitis*

## Abstract

This paper focuses on the creation of an in vitro collection of grapevine hybrids from the breeding program of the Kazakh Scientific Research Institute of Fruit Growing and Viticulture and investigates the presence of *Plasmopara viticola* resistance mediated by *Rpv3* and *Rpv12* loci. We looked at the optimization of in vitro establishment using either shoots taken directly from field-grown plants or from budwood cuttings forced indoors. We further screened for the presence of endophyte contamination in the initiated explants and optimized the multiplication stage. Finally, the presence of the resistance loci against *P. viticola* was studied. The shoots initiated from the field-sourced explants were the more effective method of providing plant sources for in vitro initiation once all plant accessions met the goal of in vitro establishment. The concentration of phytohormones and the acidity of the culture medium have a great effect on the multiplication rate and the quality of in vitro stock cultures. Out of 17 grapevine accessions, 16 showed the presence of single or combined resistance loci against *P. viticola*. The grapevine accessions identified as carrying *Rpv3* and *Rpv12* alleles represent important genetic resources for disease resistance breeding programs. These accessions may further contribute to the creation of new elite cultivars of economic interest.

## 1. Introduction

Grapevines (*Vitis* spp.) are among the most economically important temperate fruit crops cultivated and consumed throughout the world. In 2022, the planted area of grapevines, globally, reached 7.3 M hectares, producing over 70 Mt of fruit, of which 47.4% were wine grapes, 45.4% were table grapes, and 8% were grapes for drying [1]. The genus *Vitis* has a rich genetic diversity of about 80 species, with many of the commercially important cultivars assigned to *Vitis vinifera* [2,3]. Other *Vitis* species, as well as *Muscadinia rotundifolia*, provide valuable genes or traits for breeding programs focused on developing new cultivars and rootstocks that are resistant/tolerant to abiotic and biotic stresses [4,5]. Conserving the genetic diversity of grape genetic resources is essential for advancing breeding programs to ensure continued access to potentially valuable traits [6,7,8]. 

In Kazakhstan, industrial viticulture began in the 1930s, and currently, grapes are grown on about 16,000 ha and have production levels of 82.7 thousand t annually [9,10]. Kazakh viticulture is characterized by a concentrated production zone in the southern regions of the republic; 98% of its grapes are grown in the Almaty, Zhambyl, and Kyzylorda regions [10,11,12,13,14]. In 2022, imports of grapes (67 thousand t) to Kazakhstan were 15 times higher than exports and amounted to USD 50 M per year [15]. In order to reduce the dependence on imported grapes, it is necessary to ensure the progressive development of the grape and wine industry by increasing the area and productivity of grape plantations within Kazakhstan. The strategies employed for these aims are the production and supply of healthy planting material for the grape industry as well as the breeding and introduction of genotypes, including our own varieties, that are resistant/tolerant to pests and diseases and are soil climate analogues [6,13,14,16,17].

A fairly wide range of grape varieties allows for the breeding and introduction of modern highly productive varieties. Kazakhstan has a very promising level of genetic diversity in wine grapes (*V. vinifera*) and their wild relatives, including both economically and culturally valuable species [13,17,18]. This genetic biodiversity of natural populations provides a pool of genes that can confer traits for disease resistance—in particular, the genetic basis for cultivars with improved cold resistance [13,17,18]. The breeding of novel grape genotypes using DNA markers can be an effective and practical approach to accelerate the breeding process, allowing for the combination of a number of genes that confer traits of interest, such as tolerance to biotic and abiotic stresses [19,20]. Hybridization is a common method used to create new, improved grape varieties [21]. The process begins with the careful selection of both parents based on the desired traits to be incorporated into the next generation of vines [20]. The accurate characterization of plant accesses is a critical factor in the identification of suitable parents for breeding crosses [22,23,24]. Molecular methods enhance the accuracy and speed of breeding processes and are important tools for identifying and mapping resistance genes in grape breeding programs [17,25], but they have not been widely used for grapes in Kazakhstan. 

*Plasmopara viticola* [(Berk. and Curt.) Berl. and de Toni], the causal agent of downy mildew, is one of the most damaging pathogens in viticulture worldwide [26]. Grape production systems rely heavily on fungicide use due to the high susceptibility of *V. vinifera* cultivars to downy mildew. Under favorable humidity and temperature conditions, downy mildew causes significant production losses by infecting young, tender green leaf, twig, and fruit tissues [26,27,28,29]. Considerable efforts have been made internationally to identify genetic sources of resistance to downy mildew in order to limit the damage caused by this pathogen. Grapevine breeding therefore aims to introduce genetic loci that mediate resistance to *P. viticola* from *Vitis* wild species into the next generation of grapevines [28,30,31,32,33]. To date, 27 loci of resistance to *P. viticola* (*Rpv1*-*Rpv27*) have been identified. The *Rpv3* and *Rpv12* loci, derived from North American, Eurasian, and Asian grapevines (*Vitis* spp.), respectively, are the most widely used *Rpvs* for resistance to *P. viticola* and are currently of relevance in viticultural breeding programs [34,35,36,37,38]. Resistance to downy mildew is thought to involve more than one gene [36,37,38,39]. Therefore, to breed mildew-resistant grape varieties, it is highly desirable to combine as many resistance genes as possible, known as pyramiding, to provide not only effective but also durable resistance. DNA-marker-assisted selection is a powerful tool for plant breeders [36,39,40]. Combining multiple resistance genes into one genotype makes it possible to create highly resistant progeny, and the use of DNA markers accelerates this process [40,41,42,43,44,45]. 

This paper focuses on the creation of an in vitro collection for the safe storage of grapevine hybrids from the breeding program of the Kazakh Scientific Research Institute of Fruit Growing and Viticulture (KSRIFGV) and investigates the presence of *P. viticola* resistance mediated by *Rpv3* and *Rpv12* in F1 progenies derived from crosses between North American, European, and Asian grapevines. Once micropropagation systems are established, progeny showing resistance to *P. viticola* diseases will be in vitro propagated and subjected to agronomic evaluation in the field. In addition, the development of successful micropropagation systems is critical for further efforts to cryopreserve these plant accessions. These initiatives will greatly support the progressive development of viticulture in Kazakhstan. Another benefit of these selected progenies obtained through *P. viticola* resistance is a significant reduction in pesticide applications. This will reduce production costs, increase profits, and protect the environment and farmers.

## 2. Results and Discussion

### 2.1. In Vitro Establishment of Grapevine Accessions 

The in vitro propagation of grapevines can be divided into four stages: establishment, shoot multiplication, the rooting of micro-shoots, and the acclimatization (transfer from in vitro to ex vitro conditions) of in vitro plantlets [46,47]. The initial stage of micropropagation procedures is the most critical step in plant tissue culture [48,49]. A successful protocol is influenced by several parameters, such as the selection of the right explant, proper sterilization, and the inhibition of any hypersensitivity of the explants [46,47,48,49,50,51,52]. In this study, 18 grapevine accessions were established in vitro using either shoots taken directly from field-grown plants or from budwood cuttings forced indoors. We found that the optimal sterilization time in 0.1% mercuric chloride (HgCl_2_) for cultures initiated from shoots harvested directly from the field was 5 to 7 min, resulting in moderate viable culture efficiencies averaging 17% to 21% across genotypes, respectively (Table 1). While longer exposure to mercuric chloride reduced the number of infected shoots, it increased necrosis and significantly reduced the number of viable shoots, leaving 5 out of 18 accessions without viable shoots after 10 min of treatment (Table 1). Overall, the V-7/17, KV-2/9, and VII-6/72 accessions responded less positively to the HgCl_2_ treatment than did the other accessions. For the initiations using shoots harvested directly from the field, the highest percentage of viable shoots, 40%, was obtained in accessions DV-10/11 and V-7/9 after 7 min of HgCl_2_ exposure (Table 1).

For the shoots that were initiated from sprouted budwood cuttings in the laboratory, the average viability levels were 25%, 12%, and 7% for 5, 7, and 10 min of HgCl_2_ exposure, respectively (Table 2). Similarly to the field-initiated shoots, there were significant differences among the accessions regarding the level of viable shoots following HgCl_2_ exposure. The shorter sterilization time used was sufficient to produce a moderate level of viable shoots, with the highest percentages of viable shoots being obtained after 5 min of exposure to HgCl_2_, with the exception of accession V-7/9, which showed 100% contamination. The accessions DV-10/11 (50% viable shoots) and III-02/22 (50% viable shoots) had the highest percentage of viable shoots (Table 2). The procedure of sprouting shoots from cuttings in the laboratory was not effective for the initiation of the accessions KV-2/9, KV-1/10, and VII-6/72; the shoots failed to regenerate regardless of the extent of HgCl_2_ treatment. While contamination was drastically reduced with prolonged exposure to HgCl_2_ (65% at 5 min and 6% at 10 min), most shoots were necrotic (10% at 5 min and 87% at 10 min) or showed no evidence of continued growth and failed to regenerate (Table 2). 

In general, the shoots initiated from the field-sourced explants, although having higher levels of contamination regardless of the HgCl_2_ exposure time used, were more effective in providing plant sources for in vitro initiation once all plant accessions had met the goal of in vitro establishment (Table 1). While the HgCl_2_ treatments appeared to be beneficial in reducing shoot contamination, we found that the shoots sprouted in the laboratory were more tender than those sourced from the field, making the plant tissue more sensitive to the surface sterilization process.

### 2.2. Screening for Endophytes Using Bacteriological Growth Media

The contamination of plant tissue cultures by different microorganisms such as bacteria and fungi is one of the major concerns in micropropagation protocols [53,54,55]. Sometimes, even apparently healthy-looking cultures may harbor endogenous contaminants (endophytes) that may not always be apparent in the early stages of tissue cultures [56,57,58]. Some plants are hosts for a wide range of endophytes, which may only arise in tissue cultures after several rounds of subcultures and/or after the cultures have undergone some stress [56,59,60]. Therefore, given the high levels of contaminants we experienced during in vitro initiation, and to minimize the risk of endophyte contamination, we further screened for the presence of these contaminants in apparently clean tissue culture-initiated explants using a specialized medium that encourages the proliferation of endogenous contaminants. On average, 45% of all the apparently clean cultures were found to harbor endophytic pathogens that had not previously been detected in the standard plant tissue culture media (Table 3, Supplementary Figure S1). The highest levels of contamination were observed in accessions VII-6/72 (73%), III-7/15 (67%), XII-17/2 (64%), and V-7/9 (60%), while the lowest levels were observed in accessions KV-2/35 (10%), DV-10/11 (15%), and IV-14/9 (17%) (Table 3). Among the accessions evaluated, the highest percentages of clean shoots were observed in KV-2/35 (90%) and DV-10/11 (85%) (Table 3). All shoots corresponding to the infected tissue were discarded as potentially contaminated. Clean cultures of accessions KV-2/35 and DV-10/11 were used for further in vitro propagation experiments.

### 2.3. In Vitro Multiplication

Plant tissue culture media have a profound effect on both the induction of adventitious shoot formation and the promotion of axillary shoot proliferation [53,62]. MS medium [63] is often used with success to grow grapevines in in vitro culture [64,65,66], and its exogenous supplementation with phytohormones serves as a tool to regulate various processes in plant tissue cultures. Auxins and cytokinins are the most commonly used phytohormones and are often used in combination [53,67,68]. It has been reported that the addition of cytokinin in combination with auxin to the multiplication medium enhanced the proliferation efficiency of grapevine shoots [65]. Auxins are involved in all plant physiological processes, including root and callus initiation and growth [67,68,69], while cytokinins affect cell division and also stimulate axillary and adventitious shoot formation and growth [62,67,68]. Gibberellic acid is used to interrupt plant dormancy, as well as to stimulate cell division and elongate shoots [70]. The combination of cytokinin and gibberellic acid has also been shown to stimulate shoot proliferation in grapevine cultures [71]. Herein, we focused on finding an ideal phytohormone balance in an MS-based medium for grapevine in vitro propagation. Clean cultures of the accessions KV-2/35 and DV-10/11 were used for our in vitro propagation experiments using ten different MS-based media supplemented with different combinations of phytohormones. Our input parameters included different concentrations of 6-benzylaminopurine (BAP), indolylbutyric acid (IBA), gibberellic acid (GA), and thidiazuron (TDZ) at different pH levels. We also included Plant Preservative Mixture^TM^ (PPM) in a medium to control for any endophyte escape and to check for any negative impact on the growth of the in vitro cultures.

During the multiplication phase, tissue culture plants are multiplied to produce a sufficient number of progeny plants. Shoot multiplication is achieved by promoting axillary shoot growth or the induction of adventitious shoots. The effectiveness of a propagation protocol depends largely on the rate of shoot multiplication [52]. The shoot multiplication rate (MR) reflects the number of new progeny plants produced from a single explant [56,72]. We found that the concentration of phytohormones and the acidity of the culture medium have a great effect on the MR and the quality of in vitro stock cultures (Table 4). MS basal medium supplemented with 1 mg L^−1^ BAP, 0.1 mg L^−1^ IBA, and 0.1 mg L^−1^ GA at a pH of 5.7 (medium no. 7) resulted in the highest MR for both the DV-10/11 and KV-2/35 accessions, producing 4.4 and 4.1 shoots per initial explant, respectively (Table 4). The cultures grown on medium no. 7 exhibited leaves with an intense green color, no vitrified or necrotic shoots, and no abnormal plant growth (Supplementary Figure S2).

For both accessions, the decrease in the number of shoots produced by each initial explant occurred when the BAP concentration used was 0.5 mg L^−1^ in the presence of 0.2% PPM^TM^ (medium no. 6). There was no evidence of contamination during the propagation phase, which is an indication that the pre-screening for the presence of potential contaminants was effective. Therefore, these results suggest that it is not necessary to supplement the culture medium with PPM^TM^ during the multiplication phase. Although the use of PPM^TM^ can be effective in controlling endophyte contamination [56,73,74], it also has been shown to have a negative impact on in vitro plant growth, and the tolerance levels and response to PPM^TM^ vary greatly with species [75,76,77]. In order to evaluate its potential for controlling microbial contaminants, future research may include PPM^TM^ only during the in vitro establishment of grapevine cultures.

The hydrogen ion (pH) concentration of the medium, in addition to the type and concentration of phytohormones, is another determinant of successful plant micropropagation [62]. The pH of the culture medium affects the availability and uptake of nutrients and therefore has a direct effect on plant development and growth [78,79,80,81,82,83]. To access the effect of medium pH on grapevine shoot proliferation, we compared the same medium formulation within two pH ranges, i.e., medium no. 2 at a pH of 5.3 versus medium no. 7 at a pH of 5.7, which are commonly used in grapevine micropropagation [84,85,86,87]. The pH range used had no significant effect on the MR increase for either the DV-10/11 or KV-2/35 accessions. Although not significant, a slight increase in MR, 4.4 for DV-10/11 and 4.1 for KV-2/35, was observed when the pH of the medium was 5.7 compared to 3.9 for both the DV-10/11 and KV-2/35 accessions grown on the medium at a pH of 5.3 (Table 4). 

For the establishment of an in vitro collection at the Kazakh Scientific Research Institute of Fruit and Viticulture, cultures of the remaining grapevine crosses will be grown and propagated on medium no. 7. The development of optimized micropropagation systems, such as the one developed in this study, will further facilitate future efforts for the long-term conservation of grapevine plant genetic resources [6,88,89,90,91,92,93,94,95].

### 2.4. Presence of Plasmopara viticola Resistance Genes in Grape Accessions

Grapevines are highly susceptible to fungal infections, and *P. viticola* has long been a threat to grape production worldwide, especially in areas with relatively warm and humid climate conditions [26,96,97,98]. Considerable efforts have been made internationally to identify genetic resources for resistance to *P. viticola* [42,96,99,100,101,102,103,104]. The development of grapevines with such a level of resistance to this threat is a highly desirable and sustainable strategy to overcome this problem [26], and it would also contribute to reducing the massive use of pesticides in orchards. In order to introduce this trait into new cultivars, KSRIFGV plant breeders have made crosses between *Vitis* species known for their variable levels of resistance to *P. viticola*, with the aim of developing a new cultivar resistant to this pathogen and adapted to local growing conditions.

In grapevines, global programs to breed cultivars resistant to *P. viticola* have introduced resistance genes into many progenies since the mid-1800s [98]. Here, the presence of the resistance loci *Rpv3* and *Rpv12* against *P. viticola* was studied in the 17 grapevine accessions developed for the breeding program of the KSRIFGV and used in this study. Plant accessions with a single or combined allele linked to the resistance alleles *Rpv3* and/or *Rpv12* on one of the microsatellite markers for each locus were considered to have the resistance loci against *P. viticola*. Out of the 17 grapevine accessions, 16 showed the presence of one resistance locus (*Rpv3* or *Rpv12*) or multiple loci in combination (*Rpv3* + *Rpv12*) (Table 5, Supplementary Figure S3). None of the resistance alleles screened were found in accession KV-2/35 (Table 5, Supplementary Figure S3). This suggests that this accession either is susceptible to the disease or represents new sources of resistance against *P. viticola.*

According to the results of our PCR analysis using the microsatellite marker UDV-737, which is closely related to the *Rpv3* resistance loci, accessions DV-10/11, KII-1/29, KVI-1/10, XII-9/3, III-7/15, III-02/22, V-7/9, XI-13/90, and IV-4/74 were identified, as well as small amounts in accession XII-17/2 (Table 5, Supplementary Figure S3). The use of the UDV-305 microsatellite marker was less effective in the detection of the presence of the *Rpv3* resistance loci; its presence was only detected in accession III-02/22 (Table 5, Supplementary Figure S3). The resistance locus *Rpv12*, which was identified by the marker UDV-343, was observed in 13 accessions: KV-2/9, DV-10/11, IV-6/9, KII-1/29, KVI-1/10, XII-17/2, VII-6/72, VII-3/15, XI-14/9, III-02/22, V-7/9, XI-13/90, and KIY-1/64 (Table 5, Supplementary Figure S3). 

Both resistance alleles *Rpv3* and *Rpv12* were carried by accessions DV-10/11, KII-1/29, KVI-1/10, XII-17/2, III-02/22, V-7/9, and XI-13/90 (Table 5, Supplementary Figure S3). It is now widely accepted that the breeding of plant accessions carrying multiple disease resistance genes is a more sustainable strategy for the broad-spectrum control of pathogen strains and can also increase resistance durability [35,39,40,41,42,105,106,107,108,109]. Previous studies have demonstrated the additive effects of *P. viticola* resistance by pyramiding the *Rpv12* and *Rpv3* loci. The sporulation of *P. viticola* isolates in leaf discs of *V. vinifera* cultivars was reduced by the introgression of *Rpv12* + *Rpv3* resistance loci [109]. Likewise, in *V. amurensis*, *Rpv3* and *Rpv12* conferred significantly enhanced resistance to pathogen infection in comparison to single resistance alleles [35]. The grapevine accessions identified in this study as carrying *Rpv3* and *Rpv12* alleles represent important genetic resources for future breeding purposes in Kazakhstan. These accessions may further contribute to the creation of new elite cultivars of economic interest. Work is currently underway to propagate these accessions to plant them in the KSRIFGV experimental area, followed by an agronomic evaluation and testing for their resistance to *P. viticola* under field conditions.

## 3. Materials and Methods

### 3.1. Plant Material 

In the breeding program of the Kazakh Scientific Research Institute of Fruit Growing and Viticulture (KSRIFGV), crosses were made between North American, European, and Asian grapevines with the aim of carrying different resistance genes to *P. viticola*. This work has been carried out by the Institute’s breeders over the years, and the varieties were selected for their economically and agronomically valuable traits and included some of the most important cultivars of current or historical importance in Kazakhstan. Plants were managed and grown in the experimental field according to the regular practical recommendations of the KSRIFGV [13,14,110]. Cuttings of 18 selected hybrids of grapevines grown for three years in the experimental area of KSRIFGV in Almaty (Kazakhstan) were used as a source of plant material for in vitro initiation. Table 6 provides detailed information on the grape varieties used in this study and their background.

### 3.2. In Vitro Establishment and Maintenance of Grapevine Accessions 

**Figure 1:**
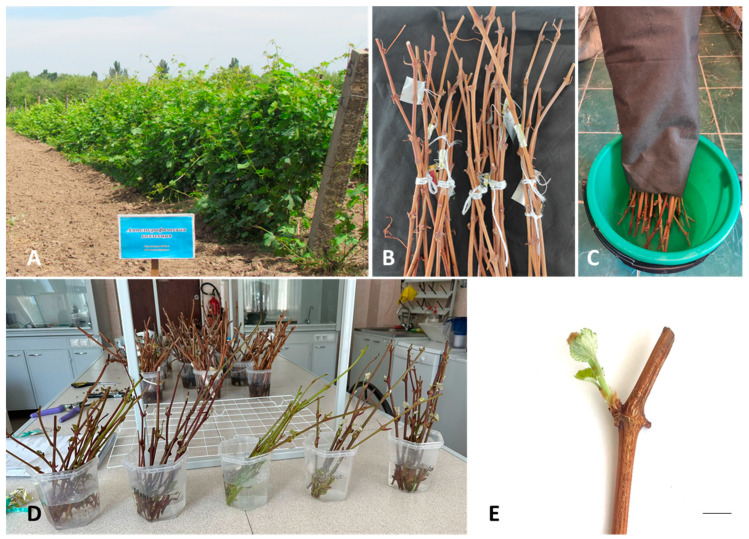
Field collection of grapevine hybrids grown in the experimental area of Kazakh Scientific Research Institute of Fruit Growing and Viticulture in Almaty (**A**). Cuttings of grapevine hybrids harvested in November (**B**) and cold-treated in a refrigerated room at 4 °C for 2 months (**C**) before moving to laboratory conditions at 24 ± 1 °C and with a photoperiod of 10 h 30 min for bud sprouting (**D**,**E**). Bar in E = 1 cm.

Cultures were initiated with shoots taken directly from the field or by sprouting new shoots from cuttings taken from dormant mother plants and sprouted in an indoor laboratory environment (Figure 1), as described below.

Cuttings, approximately 40–50 cm long containing 6–8 buds were harvested in November from three-year-old grapevines. Then, they were washed with soapy water, rinsed, and treated with 50% commercial bleach (Belizna, 5–15% sodium hypochlorite, alkaline components ≤ 5%) for 5 min. Cuttings were immediately placed in a container with water where the base of the cutting was submerged in water and the upper part of the cutting was covered with a dense, light-proof fabric to prevent premature sprouting of the buds during stratification. The stratification was performed in a refrigerator at 4 °C for 2 months, before moving to laboratory conditions (24 ± 1 °C and a of photoperiod of 10 h 30 min) for bud sprouting. The water at the base of the cuttings was replaced daily. 

Young shoots that were 1–2 cm in size containing a single bud were harvested from sprouted cuttings in the laboratory (in January) or directly from the field (harvested in April), washed with soapy water, rinsed, and treated with 0.1% mercuric chloride (HgCl_2_) solution for 5, 7, or 10 min. Afterwards, shoots were placed in test tubes of MS medium [63] containing 30 g L^−1^ sucrose, 0.5 mg L^−1^ 6-benzylaminopurine (BAP), 0.1 mg L^−1^ indolyl butyric acid (IBA), 0.1 mg L^−1^ gibberellic acid (GA), 1.5 g L^−1^ Gelrite™ (PhytoTechnology Laboratories^®^, Lenexa, KS, USA), and 4 g L^−1^ agar (PhytoTechnology Laboratories^®^, Lenexa, KS, USA) at pH of 5.7. Cultures were grown at 24 ± 1 °C during a photoperiod of 16 h of daylight with light intensity of 40 mM·m^–2^·s^–1^ with two types of OPPLE tubular fluorescent lamps: YK 21RR 16/G 21W 6500K RGB and YK 21RL 16/G 21W 4000K RGB, supplied by ElectroComplex in Corporation (Almaty, Kazakhstan). Cultures were subcultured every 3–5 weeks in glass culture vessels (237 mL) (PhytoTechnology Laboratories^®^, Lenexa, KS, USA). 

Necrotic, viable, and infected shoots were assessed visually within 28 days of in vitro initiation (Supplementary Figure S4). In addition, screening for endophytes was performed using bacteriological growth media, as plants may harbor endogenous pathogens that cannot be visually detected using standard plant tissue culture media.

### 3.3. Screening for Endophytes Using Bacteriological Growth Media 

After 4 weeks of in vitro initiation, the bases of the healthy-looking shoots (3–5 mm) were cut off and placed in Petri dishes (100 mm × 15 mm) containing bacteriological growth medium 523. Medium 523, a general medium for growth of bacteria, is composed 8 g L^−1^ casein hydrolysate (Sigma-Aldrich, St. Louis, MO, USA), 10 g L^−1^ sucrose, 4 g L^−1^ yeast extract, 2 g L^−1^ monopotassium phosphate (KH_2_PO_4_), 0.15 g L^−1^ magnesium sulfate (MgSO_4_ 7H_2_O), and 6 g L^−1^ Gelrite™ at pH of 6.9 [61]. Plates were incubated at room temperature to encourage proliferation of endogenous pathogens [56]. Therefore, cloudiness of the medium or growth of colonies on medium 523 after 2 weeks indicates contamination of the accessions. Plants showing contamination were discarded and the percentages of clean and infected shoots were recorded. Healthy looking shoots were used for further propagation experiments.

### 3.4. In Vitro Multiplication

DV-10/11 and KV-2/35 accessions were used to optimize the culture medium during the in vitro multiplication phase. Nodal sections were obtained from 6-week-old successfully initiated in vitro plants and placed in glass culture vessels (237 mL) (PhytoTechnology Laboratories^®^, Lenexa, KS, USA) with the 10 media formulations (MS-based formulations) described in Table 7 with a density of 5 nodal sections (1–2 cm) per vessel. Cultures were grown under the same conditions as the initiation phase. 

The multiplication rate (MR) was calculated in shoots grown for 5 weeks on one of the ten culture media. Cultures were subcultured two to three times every 5 weeks on the same medium formulation. MR was determined using the formula: MR = a/bc, where a is the number of new shoots, b is the number of initial shoots, and c is the number of subcultures. 

### 3.5. Testing Grape Hybrids for the Presence of Plasmopara viticola Resistance Genes

#### 3.5.1. DNA Extraction

In vitro collection of 17 grapevine hybrids grown for 5 weeks provided material for screening for the presence of fungal resistance genes. DNA was extracted using a modified cetyltrimethyl ammonium bromide procedure (CTAB), as described by Doyle and Doyle [112]. Frozen leaves (2–4 cm^2^) were ground in ceramic mortars with the addition of 1.5 mL of CTBA extraction buffer (2% CTAB, 2% PVP, 1.4 M NaCl, 20 mM EDTA, 100 mM Tris-HCl (pH of 8.0), 2% β-MET), heated to 65 °C. Further, 2 mL of 10 mg mL^−1^ RNase was added to the sample with incubation at 65 °C for 15 min. After which, an equal volume of chloroform was added to the samples, followed by shaking for 5 min and centrifugation for 10 min at 14,000 rpm. The upper phase was transferred to a new 2 mL centrifuge tube, and DNA was deposited in 2/3 of the volume of isopropanol. After incubation for 5 min at room temperature, the samples were centrifuged, in the same manner as before, and washed with 70% ethanol before being centrifuged again. The sediment was dried in the air. Next, the DNA was dissolved in 150 µL of sterile deionized water and re-precipitated with an equal volume of 4M NaCl and two volumes of 96% ethanol. After incubation at 20 °C for 15 min, the samples were centrifuged for 15 min at 13,000 rpm, and the precipitate was washed with 70% ethanol and air-dried, after which it was dissolved in 100 µL of deionized sterile water.

#### 3.5.2. PCR Analysis

PCR analysis of 17 grapevine DNA preparations was carried out using Thermo Fisher reagents. For the reaction (20 µL), 0.5 µL of a DNA preparation pre-diluted with 1:10 water, 2 pmol of each primer, 1x buffer for Taq polymerase, 25 mM MgCl_2_, 25 mM dNTP, and 0.2 µL of Taq polymerase (0.5 u) (Thermo Fisher Scientific Inc., Vilnius, Lithuania) were used. Specific primers Giop F (TCCTGCAATTCGCATTACGT) and Giop R (GGTTGCAGCTAATGGATTCCTA) were used for investigating the presence of resistance genes (*Rpv1* and *Rpv3*) to *Plasmopara viticola* in grapevine accessions derived from the crosses [113]. The following set of associated SSR markers were used: UDV-737 and UDV-305 for *Rpv3* and UDV-343 for *Rpv12*. The following program was used for the GeneAmp PCR System 9700 amplifier (“Applied Biosystems”, Waltham, MA, USA): 5 min at 94 °C—1 cycle; 30 s at 94 °C, 30 s at 57 °C, and 1 min at 72 °C—30 cycles; and 5 min at 72 °C—1 cycle. The reaction products were analyzed by electrophoresis using 1.2% agarose gel in 0.5 x TBE (Tris, borate, EDTA). The amplified fragments were visualized using the QUANTUM-CX5/EPI UV-WL gel electrophoresis system (Vilber Smart Imaging, Collégien, France)

### 3.6. Statistical Analysis 

A minimum of 10 shoots were used per replicate with three replicates per treatment in a randomized block design during the in vitro initiation experiments. The total number of shoots indexed using bacterial growth medium 523 ranged from 15 to 30, depending on the availability of samples obtained in each accession after initiation. The in vitro multiplication experiment was performed with two replicates of 15 shoots for each treatment. Means and standard error (SE) were calculated across experimental replicates and analyzed using analysis of variance, and Tukey’s mean separation test was performed. *p* ≤ 0.05 was considered significantly different. All analyses were performed using SYSTAT 12.0 software package [114].

## 4. Conclusions

Our study established and safely stored an in vitro collection of 18 grapevine hybrids developed in the breeding program of the KSRIFGV, as well as identified 16 accessions with the presence of *P. viticola* resistance mediated by *Rpv3* and *Rpv12* loci. The availability and safe storage of these genetic resources are essential for future advances in breeding programs to develop new elite cultivars of economic interest in Kazakhstan. Furthermore, optimized micropropagation systems, such as the one developed in this study, will further facilitate future efforts for the long-term conservation of grapevine plant genetic resources. 

## Figures and Tables

**Table 1:** Effects of different durations of surface sterilization with mercuric chloride (HgCl_2_) on shoots taken directly from the field.

Plant Accession	HgCl_2_ Exposure Duration (min)																	
	5						7						10					
	Infected		Necrotic		Viable		Infected		Necrotic		Viable		Infected		Necrotic		Viable	
IV-4/74	65 ± 9	ab	5 ± 5	d	30 ± 17	bc	70 ± 17	ab	10 ± 5	cd	20 ± 10	c	35 ± 13	bc	50 ± 18	b	15 ± 9	cd
X-13/90	55 ± 14	b	20 ± 17	cd	25 ± 20	bc	65 ± 9	ab	20 ± 18	c	15 ± 9	cd	56 ± 14	b	44 ± 10	bc	0 ± 0	d
IV-6/9	87 ± 5	ab	7 ± 3	d	7 ± 3	d	60 ± 19	ab	15 ± 17	cd	25 ± 20	bc	50 ± 14	b	50 ± 14	b	0 ± 0	d
IV-14/9	50 ± 14	b	5 ± 5	d	5 ± 5	d	65 ± 11	ab	15 ± 17	cd	20 ± 18	c	50 ± 14	b	45 ± 13	bc	5 ± 5	d
V-7/17	75 ± 17	ab	15 ± 9	cd	10 ± 5	cd	70 ± 17	ab	20 ± 17	bc	10 ± 0	cd	40 ± 9	bc	60 ± 14	b	0 ± 0	d
DV-10/11	70 ± 12	a	5 ± 5	d	25 ± 10	bc	35 ± 13	bc	25 ± 10	bc	40 ± 9	b	30 ± 22	bc	40 ± 5	b	30 ± 14	bc
KV-2/35	80 ± 17	ab	0 ± 0	d	20 ± 18	c	70 ± 10	ab	20 ± 10	cd	10 ± 9	cd	45 ± 13	bc	50 ± 14	b	5 ± 5	d
III-02/22	65 ± 9	ab	5 ± 5	d	30 ± 17	bc	50 ± 14	b	30 ± 17	bc	20 ± 18	c	37 ± 15	bc	53 ± 12	b	10 ± 4	cd
KV-2/9	80 ± 17	ab	20 ± 18	cd	0 ± 0	d	75 ±14	ab	10 ± 4	cd	15 ± 9	cd	55 ± 14	b	45 ± 14	bc	0 ± 0	d
III-7/15	70 ± 14	ab	10 ± 4	cd	20 ± 12	c	65 ±13	ab	20 ± 10	c	15 ± 10	cd	35 ± 14	bc	60 ± 14	b	5 ± 5	d
KV-1/10	80 ± 14	ab	7 ± 3	c	13 ± 9	cd	60 ± 15	b	20 ± 10	c	20 ± 10	c	40 ± 10	bc	55 ± 13	b	5 ± 5	d
KII-1/29	87 ± 5	a	0 ± 0	d	13 ± 13	cd	70 ± 8	a	5 ± 5	d	25 ± 16	bc	35 ± 14	bc	50 ± 14	b	15 ± 9	cd
VIII-3/45	90 ± 12	a	0 ± 0	d	10 ± 9	cd	60 ± 23	ab	20 ± 8	cd	20 ± 17	c	35 ± 13	bc	60 ± 15	b	5 ± 5	d
V-7/9	90 ± 7	a	10 ± 9	cd	0 ± 0	d	60 ± 15	b	0 ± 0	d	40 ± 5	b	50 ± 14	b	45 ± 22	bc	5 ± 5	d
XII-17/2	60 ± 5	b	10 ± 17	cd	25 ± 15	bc	60 ± 12	b	35 ± 13	bc	5 ± 5	d	32 ± 16	bc	58 ± 15	b	10 ± 4	cd
XII-9/3	60 ± 15	b	10 ± 5	c	30 ± 17	bc	50 ± 14	b	15 ± 9	cd	35 ± 9	bc	25 ± 20	bc	45 ± 14	bc	30 ± 17	bc
VII-6/72	90 ± 8	a	0 ± 0	d	10 ± 9	cd	45 ± 10	bc	45 ± 10	bc	10 ± 9	cd	45 ± 13	bc	55 ± 13	b	0 ± 0	d
KIV-1/64	75 ± 15	ab	10 ± 6	cd	15 ± 9	cd	65 ± 9	ab	10 ± 4	cd	25 ± 20	bc	50 ± 14	b	45 ± 13	bc	5 ± 5	d
Mean	75 ± 12		8 ± 6		17 ± 11		61 ± 10		18 ± 11		21 ± 11		41 ± 9		51 ± 6		8 ± 8	

Data represent mean ± SE. Values followed by different letters within each section were significantly different at *p* ≤ 0.05 using Tukey’s mean separation test. HgCl_2_, mercuric chloride.

**Table 2:** Effects of different durations of surface sterilization with mercuric chloride (HgCl_2_) on shoots sprouted in an indoor laboratory environment.

Plant Accession	HgCl_2_ Exposure Duration (min)																	
	5						7						10					
	Infected		Necrotic		Viable		Infected		Necrotic		Viable		Infected		Necrotic		Viable	
IV-4/74	60 ± 19	ab	0 ± 0	d	40 ± 10	bc	30 ± 21	bc	60 ± 15	b	10 ± 9	cd	20 ± 17	c	80 ± 11	a	0 ± 0	d
X-13/90	50 ± 14	b	20 ± 18	c	30 ± 14	bc	25 ± 20	bc	67 ± 17	ab	8 ± 5	cd	9 ± 6	cd	82 ± 12	a	9 ± 6	cd
IV-6/9	50 ± 14	b	0 ± 0	d	10 ± 10	cd	30 ±22	bc	80 ± 12	a	0 ± 0	d	0 ± 0	d	100 ± 0	a	0 ± 0	d
IV-14/9	80 ± 17	ab	10 ± 12	cd	10 ± 12	cd	30 ±22	bc	70 ± 10	ab	0 ± 0	d	0 ± 0	d	90 ± 16	a	10 ± 14	cd
V-7/17	40 ± 9	bc	20 ± 18	c	40 ± 21	bc	8 ± 7	cd	83 ± 14	a	8 ± 11	cd	8 ± 8	cd	83 ± 4	a	8 ± 8	cd
DV-10/11	40 ± 5	bc	10 ± 3	cd	50 ± 21	b	0 ± 0	d	60 ± 10	b	40 ± 23	bc	0 ± 0	d	83 ± 14	a	17 ± 7	cd
KV-2/35	30 ± 14	bc	30 ± 14	bc	40 ± 22	bc	8 ± 13	cd	80 ± 16	a	15 ± 7	cd	0 ± 0	d	91 ± 17	a	9 ± 15	cd
III-02/22	40 ± 20	bc	10 ± 12	cd	50 ± 20	b	0 ± 0	d	58 ± 24	b	42 ± 18	bc	0 ± 0	d	83 ± 14	a	8 ± 7	cd
KV-2/9	65 ± 16	ab	35 ± 21	bc	0 ± 0	d	14 ± 17	cd	86 ± 14	a	0 ± 0	d	10 ± 16	cd	90 ± 17	a	0 ± 0	d
III-7/15	50 ± 14	b	10 ± 10	cd	40 ± 22	bc	20 ± 13	c	67 ± 20	ab	13 ± 7	cd	8 ± 8	cd	92 ± 9	a	0 ± 0	d
KV-1/10	90 ±17	a	10 ± 8	cd	0 ± 0	d	20 ± 10	c	80 ± 12	a	0 ± 0	d	10 ± 3	cd	90 ± 16	a	0 ± 0	d
KII-1/29	75 ± 12	a	0 ± 0	d	25 ± 20	bc	33 ± 11	bc	50 ± 20	b	17 ± 8	cd	8 ± 6	cd	83 ± 12	a	8 ± 8	cd
VIII-3/45	80 ± 12	a	0 ± 0	d	20 ± 13	c	14 ± 17	cd	79 ± 10	a	7 ± 6	cd	0 ± 0	d	92 ± 19	a	8 ± 6	cd
V-7/9	100 ± 0	a	0 ± 0	d	0 ± 0	d	20 ± 18	c	70 ± 13	ab	10 ± 9	cd	0 ± 0	d	90 ± 17	a	10 ± 5	cd
XII-17/2	57 ± 14	b	14 ± 8	cd	29 ± 13	bc	0 ± 0	d	79 ± 20	a	21 ± 7	c	0 ± 0	d	92 ± 14	a	8 ±12	cd
XII-9/3	50 ± 9	b	10 ± 7	cd	40 ± 18	bc	10 ± 10	cd	60 ± 15	b	30 ± 20	bc	9 ± 9	cd	64 ± 17	ab	27 ± 20	bc
VII-6/72	100 ± 0	a	0 ± 0	d	0 ± 0	d	26 ± 5	bc	75 ± 22	a	0 ± 0	d	8 ± 8	cd	92 ± 14	a	0 ± 0	d
KIV-1/64	90 ± 10	a	0 ± 0	d	10 ± 10	cd	30 ± 14	bc	70 ± 12	ab	0 ± 0	d	10 ± 10	cd	90 ± 17	a	0 ± 0	d
Mean	65 ± 22		10 ± 10		25 ± 18		17 ± 11		71 ± 10		12 ± 11		6 ± 5		87 ± 7		7 ± 5	

Values followed by different letters were significantly different at *p* ≤ 0.05 using Tukey’s mean separation test. HgCl_2_, mercuric chloride.

**Table 3:** Percentage of infected and clean shoots of in vitro grapevines grown for 4 weeks and indexed using the bacterial growth medium 523.

Plant Accession	Shoots				*N*
	Infected (%)		Clean (%)		
IV-4/74	50 ± 9	b	50 ± 14	b	28
X-13/90	59 ± 14	b	41 ± 6	b	27
IV-6/9	45 ± 17	bc	55 ± 24	b	20
IV-14/9	17 ± 7	cd	83 ± 18	ab	24
V-7/17	39 ± 14	bc	61 ± 14	b	18
DV-10/11	15 ± 8	cd	85 ± 15	a	30
KV-2/35	10 ± 6.	cd	90 ± 7	a	38
III-02/22	41 ± 12	bc	59 ± 14	b	30
KV-2/9	33 ± 14	bc	67 ± 10	ab	19
III-7/15	67 ± 14	ab	33 ± 13	bc	27
KV-1/10	56 ± 16	b	44 ± 12	bc	16
KII-1/29	50 ± 12	b	50 ± 18	b	26
VIII-3/45	44 ± 17	bc	56 ± 14	b	18
V-7/9	60 ± 14	ab	40 ± 5	b	20
XII-17/2	64 ± 13	ab	36 ± 8	bc	25
XII-9/3	27 ± 14	bc	73 ± 6	ab	27
VII-6/72	73 ± 12	ab	27 ± 20	bc	15
KIV-1/64	63 ± 14	ab	37 ± 15	bc	16
Mean	45 ± 12		55 ± 11		

Values followed by different letters were significantly different at *p* ≤ 0.05 using Tukey’s mean separation test. N, number of shoots used for each treatment. Medium 523 is a general medium for the growth of bacteria [61].

**Table 4:** Influence of the composition of the nutrient medium on the multiplication rate of in vitro grapevines DV-10/11 and KV-2/35.

Culture Media	DV-10/11				KV-2/35			
	Number of Shoots		Multiplication Rate		Number of Shoots		Multiplication Rate	
	Initial	Regenerated			Initial	Regenerated		
1	15	50	3.3 ± 0.3	bc	15	51	3.4 ± 0.2	bc
2	15	58	3.9 ± 0.3	ab	15	58	3.9 ± 0.3	ab
3	15	53	3.5 ± 0.2	bc	15	54	3.6 ± 0.3	b
4	15	54	3.6 ± 0.3	b	15	53	3.5 ± 0.3	bc
5	15	45	3.0 ± 0.2	c	15	47	3.1 ± 0.2	c
6	15	39	2.6 ± 0.4	dc	15	30	2.0 ± 0.4	d
7	15	66	4.4 ± 0.3	a	15	62	4.1 ± 0.3	a
8	15	60	4.0 ± 0.1	ab	15	59	3.9 ± 0.1	ab
9	15	57	3.8 ± 0.1	ab	15	57	3.8 ± 0.1	ab
10	15	57	3.8 ± 0.2	ab	15	58	3.9 ± 0.2	ab
Mean	15	54	3.6 ± 0.3		15	53	3.5 ± 0.2	

Data represent mean ± SE. Values followed by different letters within each section were significantly different at *p* ≤ 0.05 using Tukey’s mean separation test.

**Table 5:** Presence of *Rpv3*- and *Rpv12*-mediated resistance to *Plasmopara viticola* in 17 grapevine accessions of the Kazakh Scientific Research Institute of Fruit Growing and Viticulture.

Plant Accession	Resistance Loci (Associated Markers)		
	*Rpv3* (UDV-737)	*Rpv3* (UDV-305)	*Rpv12* (UDV-343)
KV-2/9			+
DV-10/11	+		+
IV-6/9			+
KII-1/29	+		+
KVI-1/10	+		+
XII-17/2	+		+
VII-6/72			+
VII-3/15			+
XII-9/3	+		
XI-14/9			+
III-7/15	+		
III-02/22	+	+	+
V-7/9	+		+
XI-13/90	+		+
KIY-1/64			+
KV-2/35			
IV-4/74	+		

*Rpv3* and *Rpv12*— *Plasmopara viticola* resistance loci; UDV-737, UDV-305—microsatellite markers closely related to the resistance loci *Rpv*3; UDV-343—microsatellite marker closely related to the resistance loci *Rpv*12. + presence of resistance allele.

**Table 6:** List of grapevine accessions derived from crosses between North American, European, and Asian grapevines.

Breed Product	Female Parent		Male Parent	
	Name	Species	Name	Species
KV-2/9	Nimrang Magaracha	Hybrid of *Vitis berlandieri*, *V. rupestris* and *V. vinifera*	Cardinal	*V. vinifera*
DV-10/11	Madeleine Angevine	*V. vinifera*	Taifi rozovyy	*V. vinifera*
IV-6/9	Nimrang Magaracha	Hybrid of *V. berlandieri*, *V. rupestris* and *V. vinifera*	Muscat Almatinskiy	*V. vinifera*
KII-1/29	Nimrang Magaracha	Hybrid of *V. berlandieri*, *V. rupestris* and *V. vinifera*	Cardinal	*V. vinifera*
KVI-1/10	Nimrang Magaracha	Hybrid of *V. berlandieri*, *V. rupestris* and *V. vinifera*	Cardinal	*V. vinifera*
XII-17/2	Donskoy Skorospelyy	Hybrid of *V. vinifera and V. amurensis*	Pobeda	*V. vinifera*
VII-6/72	Nimrang Magaracha	Hybrid of *V. berlandieri*, *V. rupestris* and *V. vinifera*	Zolotistyy ranniy (Irsai Oliver)	*V. vinifera*
VII-3/15	Muscat fleur d‘Orange	*V. vinifera* linné subsp. Sativa (De Candolle) Hegi	Detskiy ranniy	*V. vinifera*
XII-9/3	Madeleine Angevine	*V. vinifera*	Pobeda	*V. vinifera*
XI-14/9	Madeleine Angevine	*V. vinifera*	Pobeda	*V. vinifera*
III-7/15	I-4|58	*V. vinifera*	Golden Chasselas + Riesling	*V. vinifera*
III-02/22	Donskoy Skorospelyy	Hybrid of *V. vinifera and V. amurensis*	Königin der weingarten	*V. vinifera* subsp. *vinifera*
V-7/9	No154	*V. vinifera*	Muscat Aleksandriyskiy	*V. vinifera*
XI-13/90	Madeleine Angevine	*V. vinifera*	Tarnau	*V. vinifera*
KIY-1/64	Nimrang Magaracha	Hybrid of *V. berlandieri*, *V. rupestris* and *V. vinifera*	Cardinal	*V. vinifera*
KV-2/35	Nimrang Magaracha	Hybrid of *V. berlandieri*, *V. rupestris* and *V. vinifera*	Cardinal	*V. vinifera*
IV-4/74	Pobeda	*V. vinifera*	Zhemchug Sabo	*V. vinifera*

**Table 7:** Composition and concentration of phytohormones, Plant Preservative Mixture^TM^, and pH of the nutrient medium during the multiplication of grapevine hybrids.

Culture Media	BAP	IBA	GA	TDZ	PPM^TM^	pH
	mg L^−1^				% (*v*/*v*)	
1	1	0.1	0.5	-	-	5.7
2	1	0.1	0.1	-	-	5.3
3	0.5	0.1	0.1	-	-	5.7
4	0.5	0.1	1	-	-	5.7
5	0.5	0.1	0.1	-	-	5.3
6	0.5	0.1	0.1	-	0.2	5.7
7	1	0.1	0.1	-	-	5.7
8	1	0.1	0.1	1	-	5.7
9	0.8	0.1	0.1	-	-	5.7
10	0.8	0.1	0.1	1	-	5.7

BAP, 6-benzylaminopurine; IBA, indolyl butyric acid; GA, gibberellic acid; pH, potential of hydrogen; PPM^TM^, Plant Preservative Mixture^TM^ (Plant Cell Technology, Washington, DC, USA) [111]; TDZ, thidiazuron. All chemicals were supplied by SPF Mediland (Almaty, Kazakhstan), unless otherwise specified.

## Data Availability

The datasets presented in the study are either included in the article or in the Supplementary Material. Further inquiries can be directed to the corresponding authors.

## Supplementary Materials

The following supporting information can be downloaded at: https://www.mdpi.com/article/10.3390/plants13081089/s1, Figure S1: Indexing in vitro grape shoots for the presence of contamination on bacteriological growth medium 523 (A); Clean cultures of grape hybrid KV-2/35 grown for three weeks on culture medium no. 7 (B); Figure S2: In vitro multiplication of grapevine accessions KV-2/35 (A) and DV-10/11 (B) on culture medium no. 7 grown for 5 weeks; Figure S3: Representative electrophoretic analysis of PCR products from micropropagated grapevine accessions to test for the presence of *Plasmopara viticola* resistance genes *Rpv3* and *Rpv12*; Figure S4: Shoots were contaminated (A), necrotic (B) or well developed (C,D). Shoots of grapevine accession DV-10/11 grown on initiation medium for 28 days.
