# Supplementary material for: In Vitro Collection for the Safe Storage of Grapevine Hybrids and Identification of the Presence of Plasmopara viticola Resistance Genes
Source: Plants (Basel). 2024 Apr 13;13(8):1089. doi: 10.3390/plants13081089 (PMC11053666; doi:10.3390/plants13081089)
Supplement: Supplementary file 1 [file plants-13-01089-s001.zip › plants-2953341-supplementary.pdf]

plants

Supplementary material

In Vitro Collection for the Safe Storage of Grapevine Hybrids and Identification of the Presence of *Plasmopara viticola* Resistance Genes

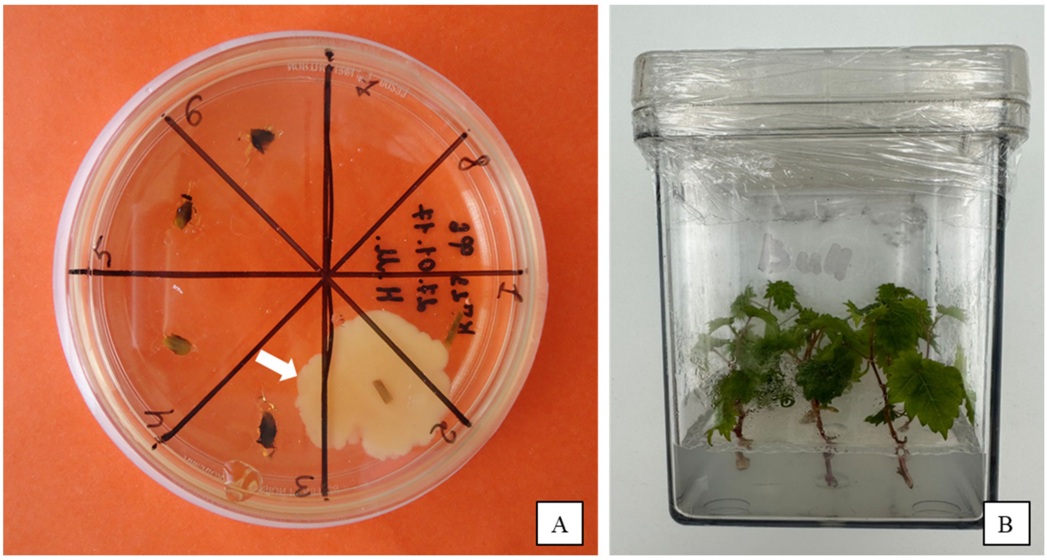

Figure S1. Indexing in vitro grape shoots for the presence of contamination on bacteriological growth medium 523 (A). Clean cultures of grape hybrid KV-2/35 grown for three weeks on culture medium no. 7 (B). White arrow in A indicates the bacterial growth on and around the grape explants and on bacteriological growth medium 523.

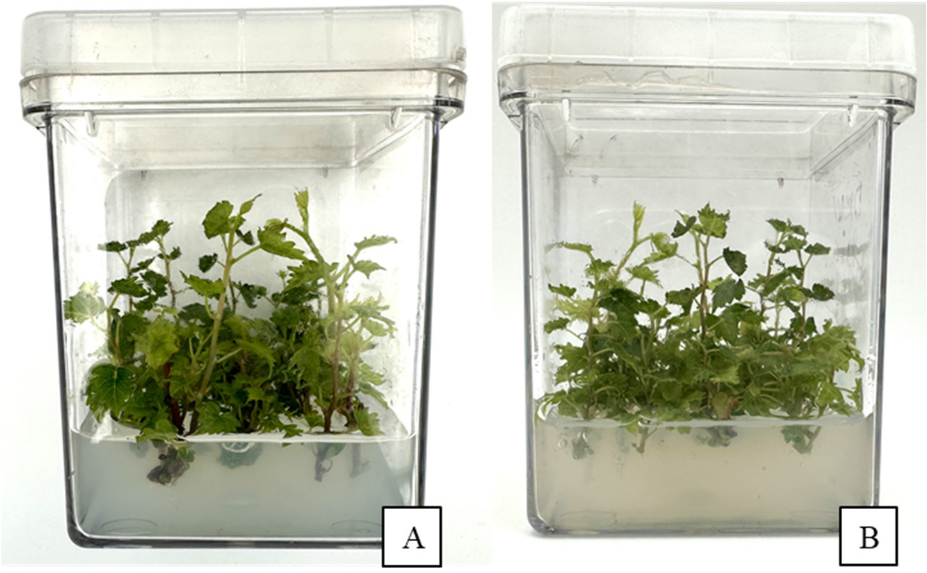

Figure S2. In vitro multiplication of grapevine accessions KV-2/35 (A) and DV-10/11 (B) on culture medium no. 7 grown for 5 weeks.

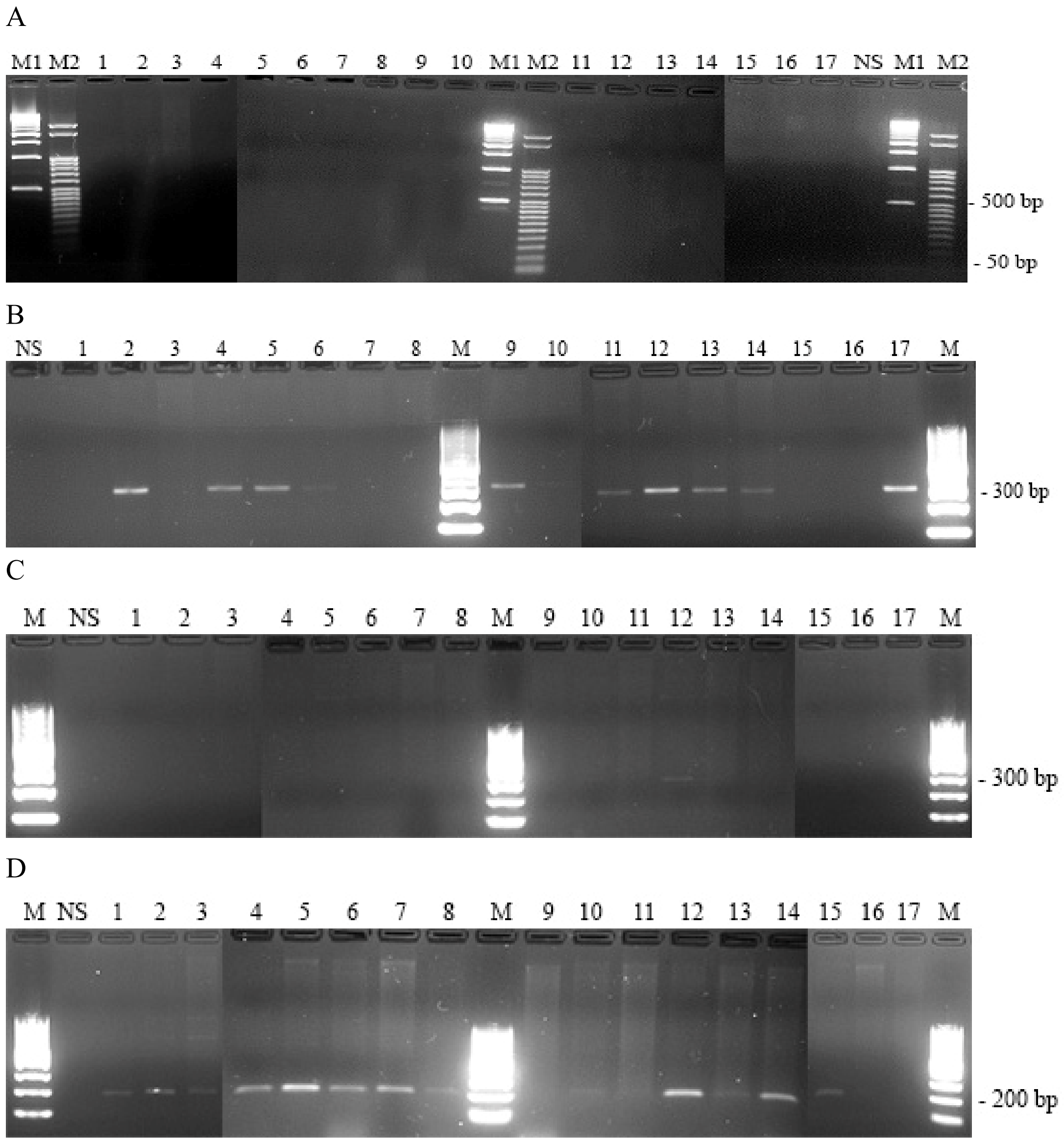

Figure S3. Representative electrophoretic analysis of PCR products from micropropagated grapevine accessions to test for the presence of *Plasmopara viticola* resistance genes *Rpv3* and *Rpv12*. (**A**) Verification of DNA markers for the presence of *P. viticola* in grapevine accessions. (**B**) PCR analysis for the presence of Rpv3 resistance loci using microsatellite marker UDV-737. (**C**) PCR analysis for the presence of Rpv3 resistance loci using microsatellite marker UDV-305. (**D**) PCR analysis for the presence of Rpv3 resistance loci using microsatellite marker UDV-343.

M1. DNA marker for electrophoresis 500 base pairs (bp) (ThermoFisher), **M2**. DNA marker for electrophoresis 50 base pairs (bp) (Sigma-Algrich®). **M.** DNA marker for electrophoresis 100 base pairs (bp) (ThermoFisher). **NS**. negative control. **1.** KV-2/9, **2.** DV-10/11, **3.** IV-6/9, **4.** KII-1/29, **5.** KVI-1/10, **6.** XII-17/2, **7.** VII-6/72, **8.** VII-3/15, **9.** XII-9/3, **10.** XI-14/9, **11.** III-7/15, **12.** III-02/22, **13.** V-7/9, **14.** XI-13/90, **15.** KIY-1/64, **16.** KV-2/35, **17.** IV-4/74.

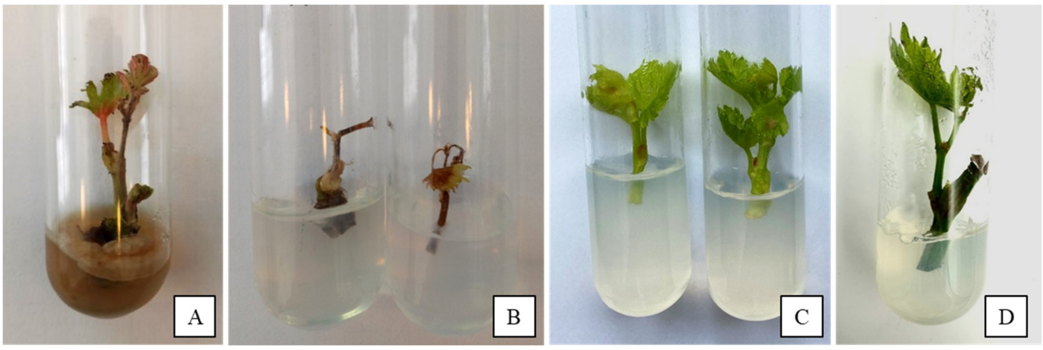

Figure S4. Shoots were contaminated (A), necrotic (B) or well-developed (C, D). Shoots of grapevine accession DV-10/11 grown on initiation medium for 28 days.
